# The Gut-Mammary Axis in Dairy Cattle: A Review of Gut Microbiota-Driven Influences on Lactation

**DOI:** 10.3390/vetsci13101107

**Published:** 2026-10-17

**Authors:** Khurshid Ahmad, Tarequl Islam, Qazi Mohammad Sajid Jamal, Supriya Srivastava, Muhammad Fazle Rabbee

**Affiliations:** 1 Department of Health Informatics, College of Applied Medical Sciences, Qassim University, Buraydah 51452, Saudi Arabia; m.quazi@qu.edu.sa (Q.M.S.J.);; 2 Department of Microbiology, Faculty of Biological Sciences, Noakhali Science and Technology University, Noakhali 3814, Bangladesh; tarequembg@gmail.com; 3 Department of Pharmacy, Faculty of Biological Sciences, Noakhali Science and Technology University, Noakhali 3814, Bangladesh; 4 Department of Biotechnology, Yeungnam University, Gyeongsan 38541, Republic of Korea

**Keywords:** gut microbiota, gut–mammary axis, mammary gland, lactation, dysbiosis

## Abstract

The gut microbiota is increasingly recognized as an important regulator of cattle health and productivity, particularly in shaping lactation performance. This review synthesizes current research on how gut microbial communities influence milk yield, composition, and lactational physiology. The diverse microbial populations in the ruminant gastrointestinal tract produce key metabolites, including short-chain fatty acids, microbial amino acids, and vitamins, that support host energy balance and nutrient utilization during lactation. We discuss the role of the gut microbiota in the gut–mammary axis and how microbial fermentation influences immune modulation, mastitis, and nutrient metabolism, all of which affect mammary gland development and milk synthesis. The review also examines how factors such as diet composition, feed additives (e.g., prebiotics, probiotics, and postbiotics), antimicrobial use, and environmental stressors alter microbiota structure and function. These changes can promote microbial resilience or dysbiosis, thereby affecting lactation either positively or negatively. Understanding these complex interactions offers promising avenues for microbiome-targeted interventions to enhance dairy performance and animal well-being. Future research should emphasize integrated multi-omics approaches to identify causal pathways and inform precision strategies for improving lactation efficiency in cattle.

## Simple Summary

Dairy cattle health and milk production depend not only on feed and animal management but also on the microorganisms living in the digestive system. These microorganisms help break down feed, release useful nutrients, support immune function, and may influence udder health and milk quality. This review examines how digestive microorganisms affect milk production, milk composition, and the risk of udder inflammation, as well as how feeding practices and microbial supplements may influence these relationships. The available evidence suggests that microbial activity in the rumen contributes to energy supply and the production of milk components. Differences in microbial communities have also been linked to changes in milk fat, protein, and other quality indicators, as well as udder health. However, many reported relationships are observational and do not prove that particular microorganisms directly cause these changes. The effects of microbial supplements also depend on the specific organisms and how they are used. A better understanding of these relationships may help researchers develop improved feeding and health-management strategies, support animal welfare, enhance milk quality and production efficiency, and promote more sustainable dairy farming.

## 1. Introduction

Lactation represents a critical metabolic period in dairy cattle, characterized by notably increased demands for nutrients, metabolic regulation, and immune defense. In recent years, researchers have highlighted gut microbiota as a central contributor to lactation through its multifaceted effects on energy metabolism, immunity, and mammary health. At the core of this interaction is microbial fermentation in the rumen, the largest stomach compartment in the cattle gut, which generates essential metabolites such as short-chain fatty acids (SCFAs) and microbial proteins. In Tibetan cattle, elevated SCFA levels have been documented within two weeks postpartum, bolstering energy supply during the early lactogenesis phase [1]. Complementing these findings, targeted probiotic supplementation with strains such as *Lactobacillus plantarum, Bifidobacterium bifidum*, and *Streptococcus thermophilus* has been shown to enhance microbial crude protein production, increase dry matter intake (DMI), and significantly improve milk yield in crossbred cows [2]. Beyond fermentative benefits, probiotics and postbiotics may influence immune responses and mammary function. Supplementation with *L. casei* and *L. plantarum* in lactating cows increased milk immunoglobulin G (IgG), lactoferrin, lysozyme, and peroxidase levels while reducing somatic cell counts (SCC), signifying improved udder immunity and a potentially lower risk of mastitis [3]. Similarly, postbiotic supplementation during the transition period enhanced nutrient digestibility, colostrum fat and protein concentrations, and milk yield through 60 days postpartum [4]. Yeast and lactic acid bacterial supplementation have also been associated with reduced concentrations of inflammatory cytokines (e.g., TNF-α, IL-1β, and IL-6) in milk, lower milk enzyme markers of blood–milk barrier damage, and alterations in the milk microbiota [5]. In late-lactation cows, shifts toward greater microbial pathway diversity, particularly in amino acid and lipid metabolism, are reflected by elevated fecal concentrations of phenylalanine, phosphocholine, and histamine, which may influence inflammation through mechanisms involving gut permeability [6].

Rumen microbiome succession during the periparturient period is also associated with production-related traits. The *Firmicutes*-to-*Bacteroidetes* (*F/B*) ratio has been proposed as a marker of gut microbial metabolism and immunoregulation in mammals [7,8]. The *F/B* ratio and specific microbial groups, such as *Prevotellaceae*, have been associated with milk fat and volume, although the strength and consistency of these associations vary across studies [9]. In addition to their roles in fermentation and immunity, gut-derived metabolites such as succinate from *Prevotella* species contribute to gut homeostasis, energy regulation, and systemic inflammation, potentially influencing host energy balance and udder health [10].

Moreover, integrative multi-omics analyses are beginning to clarify how host genetics modulate microbiome composition and lactation efficiency. A large-scale association study in Holsteins showed that rumen microbial functions, along with specific host single-nucleotide polymorphisms, may enhance milk production through metabolic pathways such as linoleic acid metabolism [11].

Taken together, these studies reveal a network of interrelated pathways through which the gut microbiota influence lactation by enhancing fermentation and feed conversion, modulating immune responses, shaping the mammary microbiome, and mediating host–microbe metabolic communication. Although previous reviews have discussed rumen microbiota in relation to fermentation, nutrient utilization, and dairy productivity, the connections between microbial metabolism, systemic immune regulation, mammary health, and lactation outcomes warrant a more integrated synthesis. This review addresses this gap by examining the gut–mammary axis in dairy cattle, linking microbial metabolites and host immune responses with mastitis, milk yield, and milk composition. It further integrates evidence on dietary and microbiome-targeted interventions, highlights limitations in establishing causal relationships, and identifies priorities for multi-omics research and precision strategies to improve lactation efficiency.

## 2. Methods

For this review, we conducted a focused literature search in PubMed, Scopus, Web of Science, and Google Scholar for studies published from 2015 to 2026, plus earlier landmark studies. Search terms included combinations of “gut microbiota,” “rumen microbiota,” “gut–mammary axis,” “dairy cattle,” “lactation,” “milk yield,” “milk composition,” “mastitis,” and “microbiome.” Studies were included if they investigated gastrointestinal microbiota, microbial metabolites, immune regulation, mammary health, lactation, or related mechanisms relevant to the gut–mammary axis. Evidence from dairy and beef cattle, buffalo calves, and mouse models was considered when relevant to these objectives. Human immunological studies were used only to provide background on established host–microbiota mechanisms in mammals, not as direct evidence of effects in cattle. Findings were interpreted according to the species and study model, with direct evidence from cattle distinguished from indirect or mechanistic evidence from other models when direct findings from dairy cattle are limited. Relevant review articles were also consulted for background and contextual interpretation.

## 3. Microbial Function on the Gut-Mammary Axis

The gut–mammary axis describes a proposed, potentially bidirectional pathway through which the gastrointestinal microbiota may influence mammary health, particularly mastitis, through microbial metabolites, immune modulation, and microbial translocation. In dairy cattle, microbial products such as LPS originating in the rumen can enter the systemic circulation and influence the mammary gland, and it has been proposed that viable microbes may also reach the mammary gland, possibly carried by immune cells [12]. This section primarily examines gut-to-mammary effects on mammary inflammation and mastitis, while acknowledging that reciprocal mammary-to-gut interactions remain less clearly characterized.

Rumen microbial imbalance, such as that observed during subacute ruminal acidosis (SARA), increases the permeability of the gastrointestinal and blood–milk barriers. This allows lipopolysaccharide (LPS) and pathogenic bacteria to translocate into the circulation and the mammary gland, thereby provoking inflammation and disrupting tight-junction integrity, reducing alkaline phosphatase (ALP) levels, and contributing to mastitis severity [13]. Comparative studies have shown that cows with mastitis exhibit significantly higher serum concentrations of LPS, TNF-α, and IL-1, which are associated with shifts in the rumen microbiota, including increased abundance of *Proteobacteria* and of the Lachnospiraceae genus *Moryella* and decreased abundance of beneficial genera such as *Prevotella* or *Olsenella* [14].

Beneficial gut bacteria produce SCFAs, particularly butyrate, which strengthen epithelial barriers and exert anti-inflammatory effects. For example, *Roseburia*, a butyrate producer, has been shown to reduce gut permeability, prevent pathogen translocation, and mitigate mastitis symptoms [15]. In contrast, SARA-induced increases in sialic acid promote Enterobacteriaceae overgrowth, resulting in gut dysbiosis, increased LPS levels, and mammary inflammation through TLR4–NF-κB/NLRP3 signaling. Interventions involving commensal bacteria or inhibitors of sialic acid release have been shown to attenuate mastitis in mice [16].

**Figure 1:**
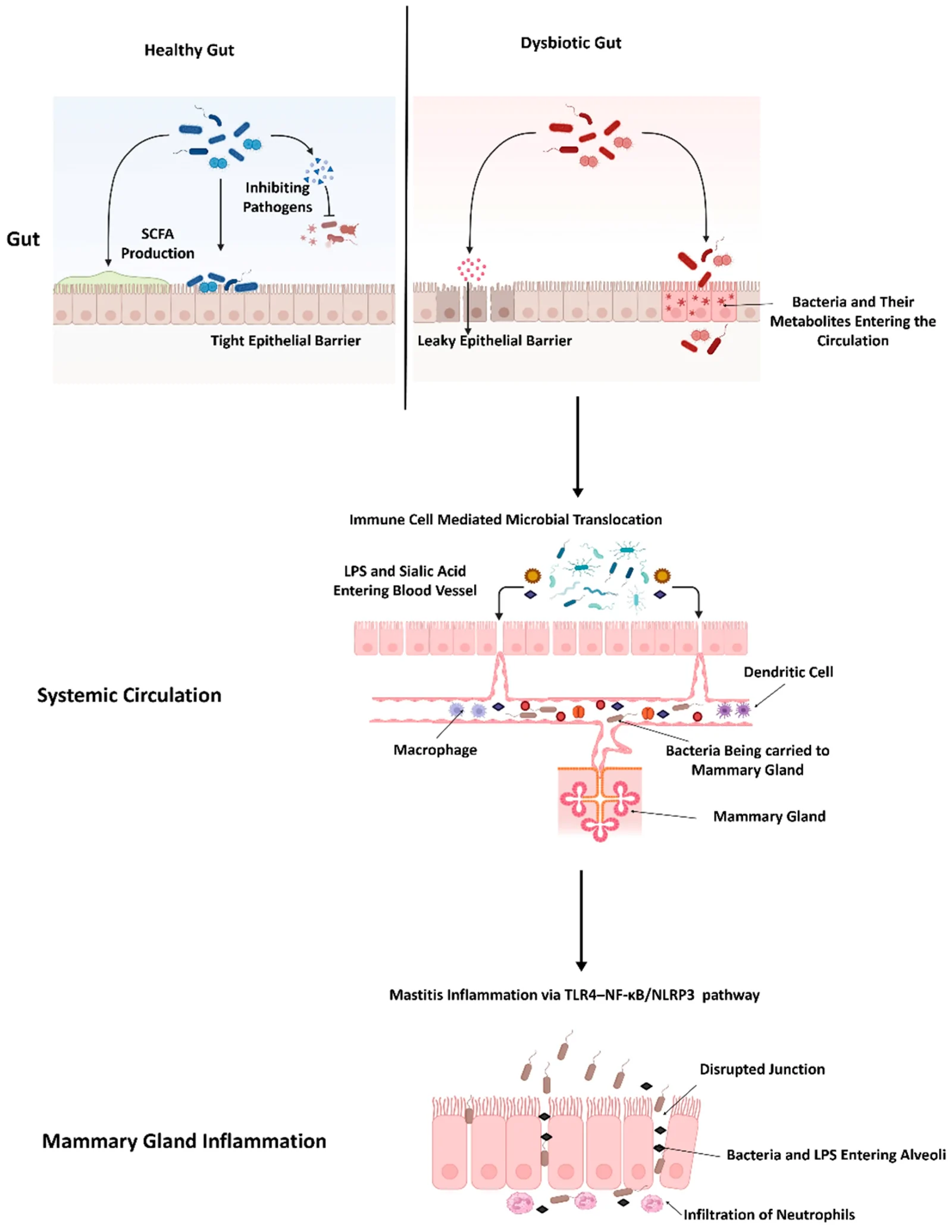
Proposed model illustrating the gut–mammary axis in dairy cattle. Gut dysbiosis promotes the translocation of LPS, pro-inflammatory metabolites (e.g., sialic acids), and, as a proposed route, immune cell-carried bacteria into the systemic circulation. These factors reach the mammary gland, where they trigger inflammation through TLR4–NF-κB signaling, impair barrier function, and increase susceptibility to mastitis. The figure was created using BioRender software v. 04.

Bacterial genera reported in both fecal and milk or blood samples suggest possible overlap between microbial communities but do not establish direct gut-to-mammary migration (Figure 1). In cows and mouse models, bacterial taxa such as *Ruminococcus*, *Peptostreptococcus*, and *Bifidobacterium* were detected in both feces and milk or blood. Moreover, oral administration of green fluorescent protein-labeled *E. coli* provided experimental evidence of gut-to-mammary translocation in mouse mastitis models [16]. It has been proposed that dendritic cells and macrophages sample luminal bacteria and carry them through the lymphatic and systemic circulation, particularly when intestinal permeability is increased [17]. Although translocation has been reported in mouse models, evidence for this cell-mediated pathway in dairy cattle remains limited; therefore, it should be regarded as a proposed mechanism rather than an established route. Demonstrating such transfer requires strain-level comparisons and appropriate controls to exclude alternative sources, including teat skin, the teat canal, environmental exposure, and laboratory contamination. Normally, the integrity of the blood–milk barrier allows for normal material exchange between blood and the mammary acinus and prevents excessive inflammatory mediators and pathogen invasions [18]. However, the permeability of the blood–milk barrier is regulated by many factors. Recently, studies in animal models have shown that the gut microbiota and its metabolites play an important role in regulating blood–milk barrier permeability by affecting tight junction protein expression [19,20], leading to the accumulation of immune cells in the mammary acinar cells and promoting the occurrence and development of mastitis.

## 4. Gut Microbiota in Immune Modulation

The gut microbiota plays a central role in shaping the immune landscape of cattle. Beyond its function in digestion and nutrient absorption, the microbiota acts like a “forgotten organ” that educates and regulates host immune responses throughout life [21,22]. In ruminants, the rumen and hindgut microbiota, including *Prevotella*, *Butyrivibrio*, *Barnesiella*, *Clostridium clusters*, *Ruminococcus*, and *Bacteroides*, contribute to fiber fermentation and the production of SCFAs such as acetate, propionate, and butyrate, which function both as energy sources and modulators of immune function [22]. Butyrate and propionate function as histone deacetylase inhibitors, facilitating the epigenetic reprogramming of immune cells, promoting regulatory T-cell (Treg) differentiation, and stimulating the production of anti-inflammatory cytokines (e.g., IL-10). These effects may contribute to a systemic tolerogenic environment that could limit excessive inflammation in the mammary gland, although this has been inferred mainly from rodent and human studies [23,24]. SCFAs, particularly butyrate, support epithelial tight-junction integrity and mucosal defense, preventing microbial translocation and endotoxemia [25]. Conversely, gut dysbiosis due to antibiotic use can deplete butyrate-producing bacteria, including members of the *Lachnospiraceae*, and impair barrier integrity; in calves, such dysbiosis was accompanied by increased methanogen abundance [26].

**Figure 2:**
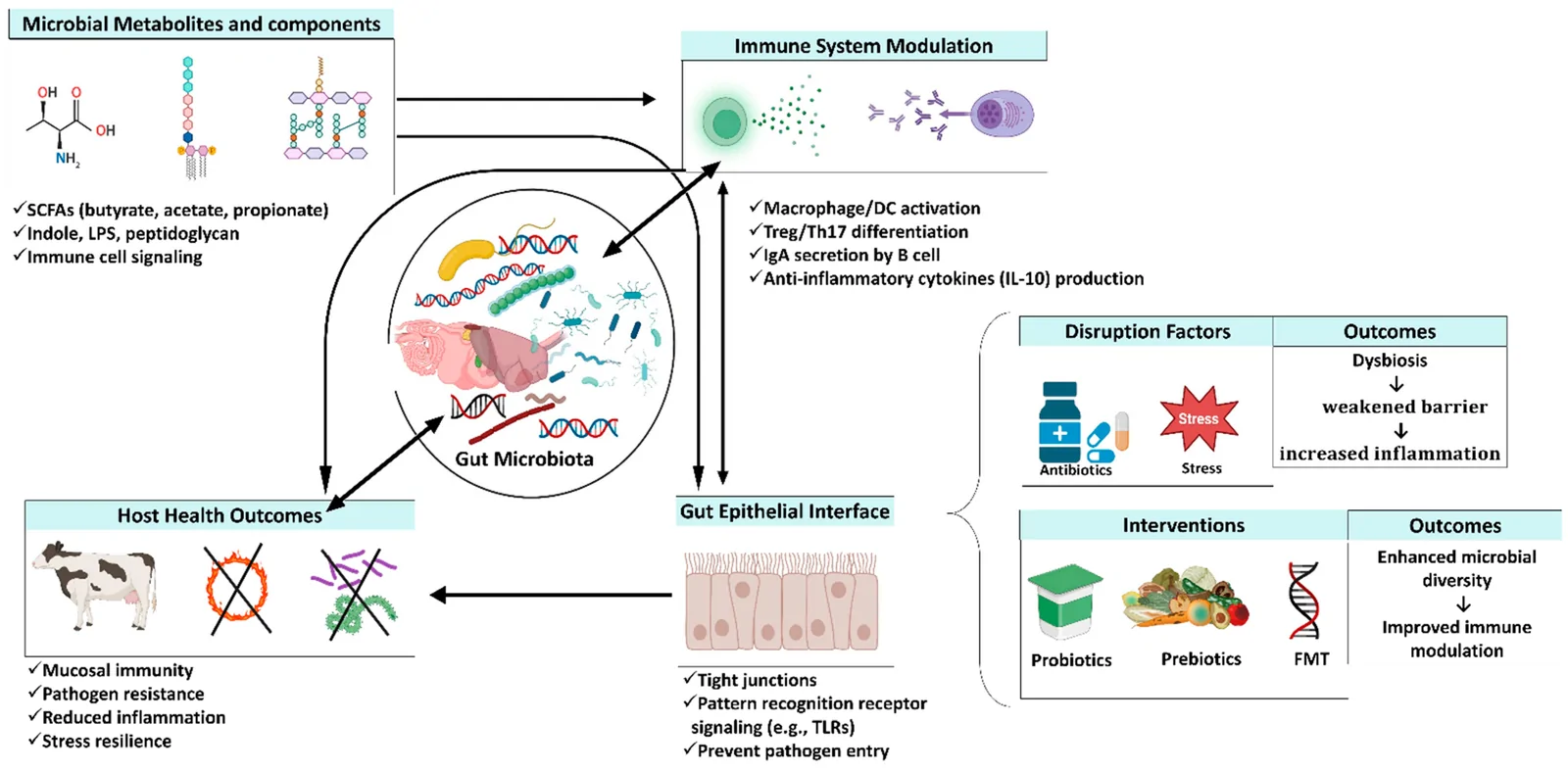
Schematic illustration of the role of the gut microbiota in immunomodulation in cattle. The figure was created using BioRender software.

*Bacteroides fragilis* and other commensal bacteria can influence mucosal immune development through microbial immunomodulatory molecules. In particular, polysaccharide A (PSA) from *B. fragilis* has been shown in mammalian experimental models, particularly mice, to promote regulatory T-cell responses and IL-10 production through TLR2-dependent mechanisms (Figure 2) [27,28]. However, direct evidence for a comparable PSA-mediated effect in cattle remains limited. In cattle, early exposure to colostrum and maternal microbes expands *Lactobacillus* and *Bifidobacterium* populations [29], decreases opportunistic pathogenic *E. coli* and *Escherichia-Shigella* genus in the colon of dairy calves [30], and enhances mucosal immunity and gut maturation [28,31], although the specific contribution of individual bacterial molecules requires further investigation. The gut microbiota interacts with mucosal immune cells and contributes to the maturation and polarization of innate and adaptive immune compartments. It has been proposed that, following this microbial education, immune cells, including tolerogenic dendritic cells, Tregs, and tolerogenic macrophages, migrate through the circulatory and lymphatic systems to distal sites, including the mammary gland, where they may modulate local immune surveillance and inflammatory responses (Figure 1); direct evidence for this in cattle remains limited. Such systemic immune education may influence the mammary gland’s capacity to respond to pathogens while limiting unnecessary inflammatory responses [32].

One essential mechanism through which gut microbes influence immunity is pathogen exclusion. Commensals outcompete pathogens for adhesion sites and nutrients, produce bacteriocins, and prime innate defenses, including mucosal antimicrobial peptide production and mucus secretion [22]. They also interact with pattern recognition receptors (PRRs), including Toll-like receptors (TLRs), thereby shaping host inflammatory tone. In cattle with high feed efficiency, enrichment of taxa such as *Muribaculaceae*, *Moryella*, *Weissella*, and *Arthrobacter* was associated with increased expression of genes such as *TLR4*, *LY96*, and *MBL2*, which are crucial for pathogen recognition and immune activation [33].

High-grain diets in dairy cows have been associated with reduced microbial diversity and increased inflammatory gene expression through the NF-κB pathway in rumen epithelium. This inflammatory response was accompanied by increased abundances of specific microbiota, including *Succinivibrionaceae* and *Ruminococcaceae*, consistent with a gut–immune link influenced by fermentative products [25]. Calves experiencing weaning stress often exhibit shifts in luminal microbiota and reductions in beneficial taxa; this may lead to decreased concentrations of anti-inflammatory cytokines, defensins, and IgA, thereby increasing disease susceptibility [34]. Supplementation with compounded probiotics in Holstein calves accelerated gut microbiome development, increased beneficial taxa, and facilitated immune maturation, including increased IgA and a balanced cytokine profile, within weeks [35].

Emerging research has also highlighted the significance of extracellular vesicles (EVs) released by gut microbiota in mediating cross-kingdom communication and delivering bioactive molecules (e.g., proteins, lipids, and RNAs) to host immune cells. Microbiota-derived EVs can regulate epithelial healing, immune cell maturation, and pathogen defense, a frontier that is yet to be extensively explored in cattle but remains important [22].

The complex interplay between microbiota-derived metabolites, including SCFAs and bile acids, and host immunity involves the modulation of dendritic cells, macrophage polarization, T-cell differentiation, epithelial turnover, and complement expression. In cattle, these interactions are reflected in associations among metabolomic profiles, PRR signaling pathways such as TLR4–NF-κB, and systemic immune gene expressions. Altogether, these findings reveal a multilayered microbiome–gut–organ axis that may influence not only the gut but also mammary, hepatic, neural, and endocrine tissues [22,25,33,34].

The liver acts as a metabolic and immunological relay by filtering gut-derived molecules and generating acute-phase reactants and inflammatory mediators that can influence the mammary environment. Through this gut-liver-mammary connection, gut microbial signals are integrated with systemic host responses that influence mammary tissue adaptation, resistance to infection and lactational output [23,36]. Table 1 summarizes the different roles of the gut microbiota in immune modulation in mammals; most entries are derived from studies in humans and rodents, and their relevance to cattle remains to be confirmed.

## 5. Gut Microbiota and Mastitis

Mounting evidence highlights a crucial gut–mammary axis through which disruption of ruminal or intestinal microbial communities may promote mastitis in dairy cows by impairing barrier integrity, promoting systemic inflammation, and facilitating the translocation of bacterial products into the udder. Cows with mastitis have been reported to exhibit marked alterations in their ruminal and fecal microbiota, characterized by enrichment of *Proteobacteria* (e.g., *Moraxella*) and of *Saccharofermentans* (*Firmicutes*) and depletion of butyrate-producing taxa (*Roseburia*, Lachnospiraceae, and *Coprococcus*) and lactic acid-producing bacteria (*Bifidobacterium* and *Lactobacillus*) [13]. *Bifidobacterium* does not produce butyrate directly; its acetate and lactate can support butyrate production by other taxa through cross-feeding, as shown mainly in the human colon [45].

These microbial alterations may have important functional consequences. In a rodent model, transplantation of ruminal microbiota from cows with mastitis induced mastitis-like phenotypes, accompanied by elevated serum LPS levels, reduced ALP activity, disruption of the gut and blood–milk barriers, and activation of inflammatory signaling pathways, including TLR4–cGAS–STING–NF-κB/NLRP3 [13]. LPS-driven endotoxemia may further impair mammary tight-junction integrity, facilitating the translocation of commensal and opportunistic bacteria such as *Enterobacteriaceae* and *Stenotrophomonas* into mammary tissue and exacerbating udder inflammation [15].

Conversely, strategies that enhance gut health may have potential for mastitis control. The butyrate-producing genus *Roseburia*, for example, has been shown to reinforce intestinal and mammary barrier integrity by limiting bacterial translocation and reducing mastitis severity in mice [15]. In mice, supplementation with calf intestinal ALP or the neuraminidase inhibitor zanamivir restored systemic ALP activity, facilitated LPS detoxification, and significantly reduced pathogen-induced mastitis [13]. These mouse findings identify potential mechanisms for reducing inflammatory cytokines and restoring blood–milk barrier integrity, but they cannot be extrapolated to therapeutic efficacy in dairy cattle without controlled trials in cows.

Probiotic supplementation has also been evaluated in cows with elevated SCC. In a 40-day trial in Holstein cows with milk SCC above 500,000 cells/mL (*n* = 6 per group), dietary yeast and lactic acid bacteria lowered milk SCC, and the combined supplement reduced milk TNF-α, IL-6, and IL-1β concentrations. Milk myeloperoxidase and N-acetyl-β-D-glucosaminidase activities, used as indirect markers of neutrophil infiltration and blood–milk barrier damage, were also reduced [5]. Clinical mastitis incidence and tight-junction proteins were not assessed.

Microbiome profiling has also identified bacterial signatures associated with mastitis. In a cross-sectional comparison of mid-lactation Holstein cows, models incorporating rumen and hindgut bacteria such as Erysipelotrichaceae, *Schwartzia*, Absconditabacteriales, and Ruminococcaceae discriminated cows with mastitis from healthy cows [46]. Because these models classified existing disease, their value for predicting future mastitis requires prospective validation. These microbial shifts have been associated with reduced metabolic capacities (e.g., vitamin B, fatty acid, and amino acid pathways) that normally support immune resilience [47].

In summary, the gut microbiota may influence bovine mastitis through three interconnected mechanisms: (1) barrier maintenance, whereby healthy, butyrate-producing microbes (e.g., *Roseburia* and *Lachnospiraceae*) support gut and mammary barrier integrity, preventing LPS and microbes from entering systemic circulation; (2) immune modulation, whereby gut dysbiosis elevates systemic LPS levels, triggers pro-inflammatory cascades, and compromises host defenses, thereby creating a mammary environment more susceptible to infection (Figure 1); and (3) therapeutic intervention, whereby restoration of microbial balance may attenuate dysbiosis-induced inflammation and improve milk quality, as shown mainly in mouse models and small trials in cows with elevated SCC (Table 2). However, functional assignments based on genus-level abundance should be interpreted cautiously, as metabolic capabilities can differ among species and strains and may vary with diet, physiological state, microbial community interactions, and host context.

## 6. Gut Microbiota on Nutrient Utilization

In adult ruminants, most microbial fermentation occurs in the rumen, where dietary carbohydrates are fermented into SCFAs, which in ruminant nutrition are also termed volatile fatty acids (VFAs), principally acetate, propionate, and butyrate. Fiber and resistant starch that escape ruminal fermentation and small-intestinal digestion are further fermented in the hindgut [54]. Studies have shown that small-intestinal digestion of rumen-escape starch (RES) in dairy cows is incomplete and variable, with reported digestion efficiencies typically ranging from approximately 50–84% of RES, depending on the intake level, starch source, and physiological state [55]. Rumen microorganisms, including *Streptococcus bovis*, *Ruminobacter amylophilus*, and *Prevotella bryantii*, produce a range of amylolytic enzymes (e.g., α-amylase and pullulanase) that catalyze the hydrolysis of starch into fermentable sugars, leading to the generation of VFAs, such as acetate, propionate, and butyrate [56]. Diets with adequate starch levels tend to favour a mixed community of amylolytic and lactic acid-producing taxa such as *Proteobacteria*, *Megasphaera elsdenii*, *Streptococcus bovis*, *Selenomonas ruminantium*, and *Prevotella bryantii,* thereby shaping metabolite production and host physiology [57]. In ruminants, microbial fermentation, predominantly in the rumen, supplies approximately 70% of the host’s energy requirements [58]. In the hindgut, *Firmicutes*, *Bacteroidetes*, *Actinobacteria*, and *Verrucomicrobia* ferment and decompose fibrous xylose and hemicellulose to meet host energy and metabolic processes, and participate in immunity [59,60].

Balanced protein diets containing both animal and plant sources are associated with microbial communities that support gut and metabolic health [61]. Ruminal microbes play a central role in protein nutrition in dairy cattle by converting dietary nitrogen, including non-protein nitrogen, into microbial protein. Unlike monogastric animals, dairy cows obtain much of their metabolizable protein from microbial protein synthesized in the reticulorumen, together with rumen-undegradable dietary protein [58,62]. Members of the Firmicutes, Bacteroidetes, and Proteobacteria contribute to this supply of VFAs, microbial protein, and vitamins [63,64].

The gut microbiota also contributes to vitamin supply and mineral availability in dairy cattle. Microorganisms in the forestomach and hindgut contribute to feed digestion, vitamin synthesis, and the absorption of minerals and other micronutrients [36,65,66]. Ruminal bacteria (members of the Bacteroidetes and Firmicutes, including *Prevotella*) produce various water-soluble B vitamins (such as cobalamin-B12). These vitamins work to improve the cow’s metabolism, energy production and milk quality. Vitamin B12 can be synthesized only by bacteria and archaea, and its ruminal synthesis requires an adequate cobalt supply. Studies have shown that microorganisms can produce more B12 if there is enough fiber in the diet [65,66,67]. A metagenomic approach reveals that microbes in the stomach and large intestine of ruminants, mainly the phyla *Bacteroidetes*, *Firmicutes*, and *Proteobacteria,* produce fat-soluble vitamin K2 (menaquinone) in the cow’s body through a ‘de novo’ process [65]. A previous study showed that a higher ruminal concentration of vitamin B12 was correlated with greater abundance of *Prevotella*, whereas a lower concentration was correlated with greater abundance of *Bacteroidetes*, *Ruminiclostridium*, and *Butyrivibrio*; the authors noted that bacterial consumption of B12 in the rumen may be as important as its production [67]. Phosphorus or other minerals in plant foods are often bound as phytate (phytic acid). Gut bacteria produce enzymes called phytases to break this bond, allowing calcium, magnesium and phosphate to be more easily absorbed into the cow’s blood [68,69]. Early lactation is characterized by negative energy balance and metabolic stress. During this period, a well-adapted gut microbiota may support nutrient absorption and has been associated with a lower risk of disorders such as ketosis and acidosis [70].

**Figure 3:**
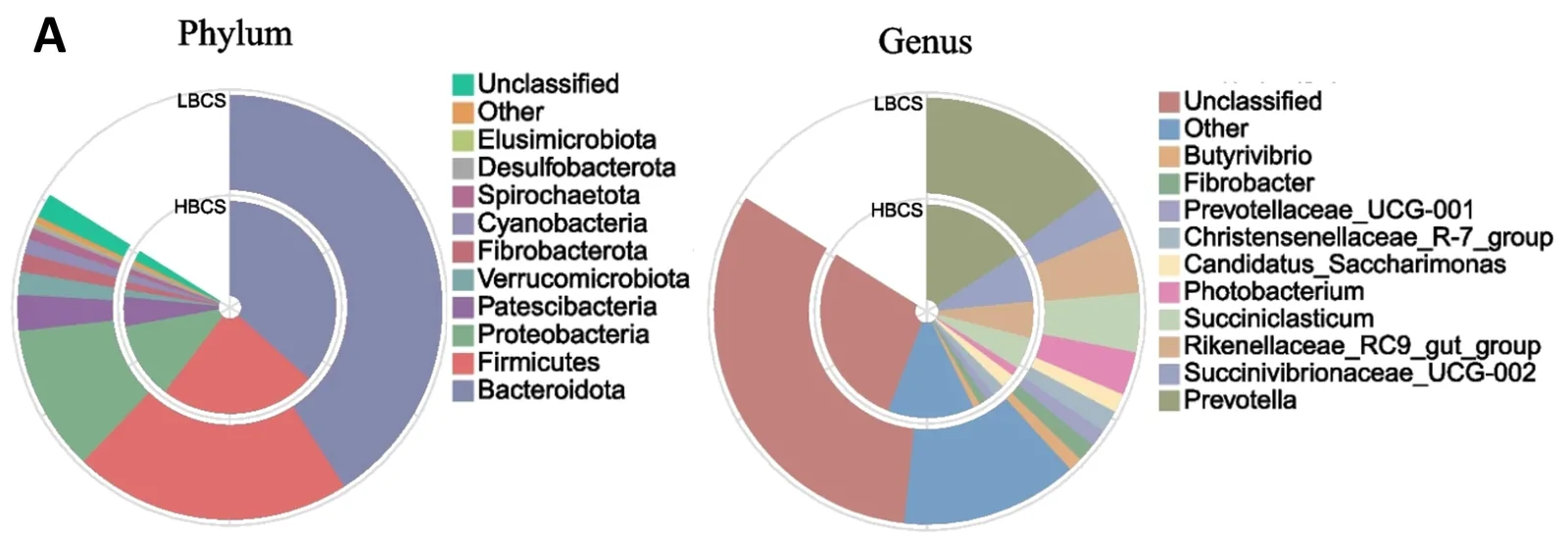

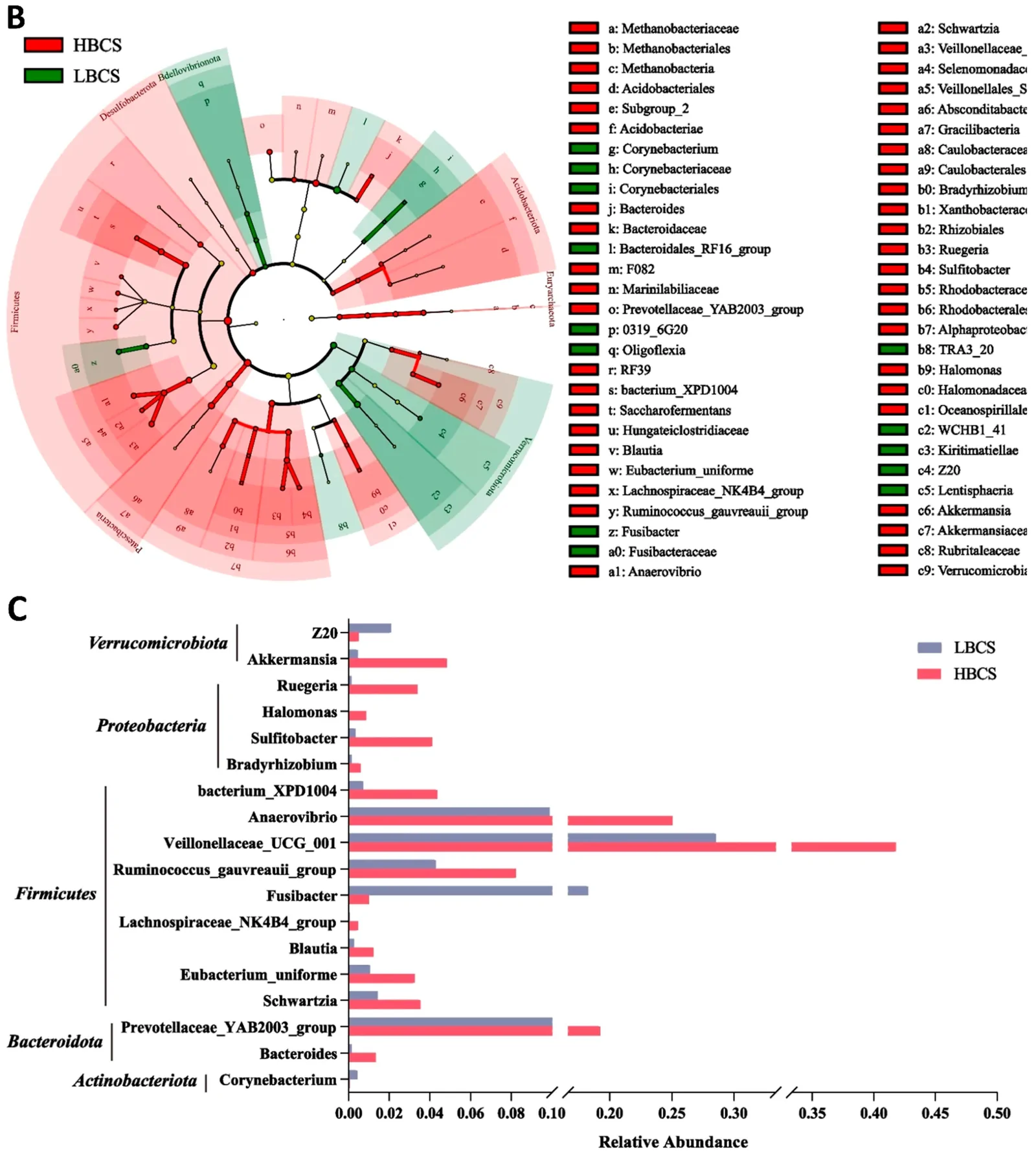

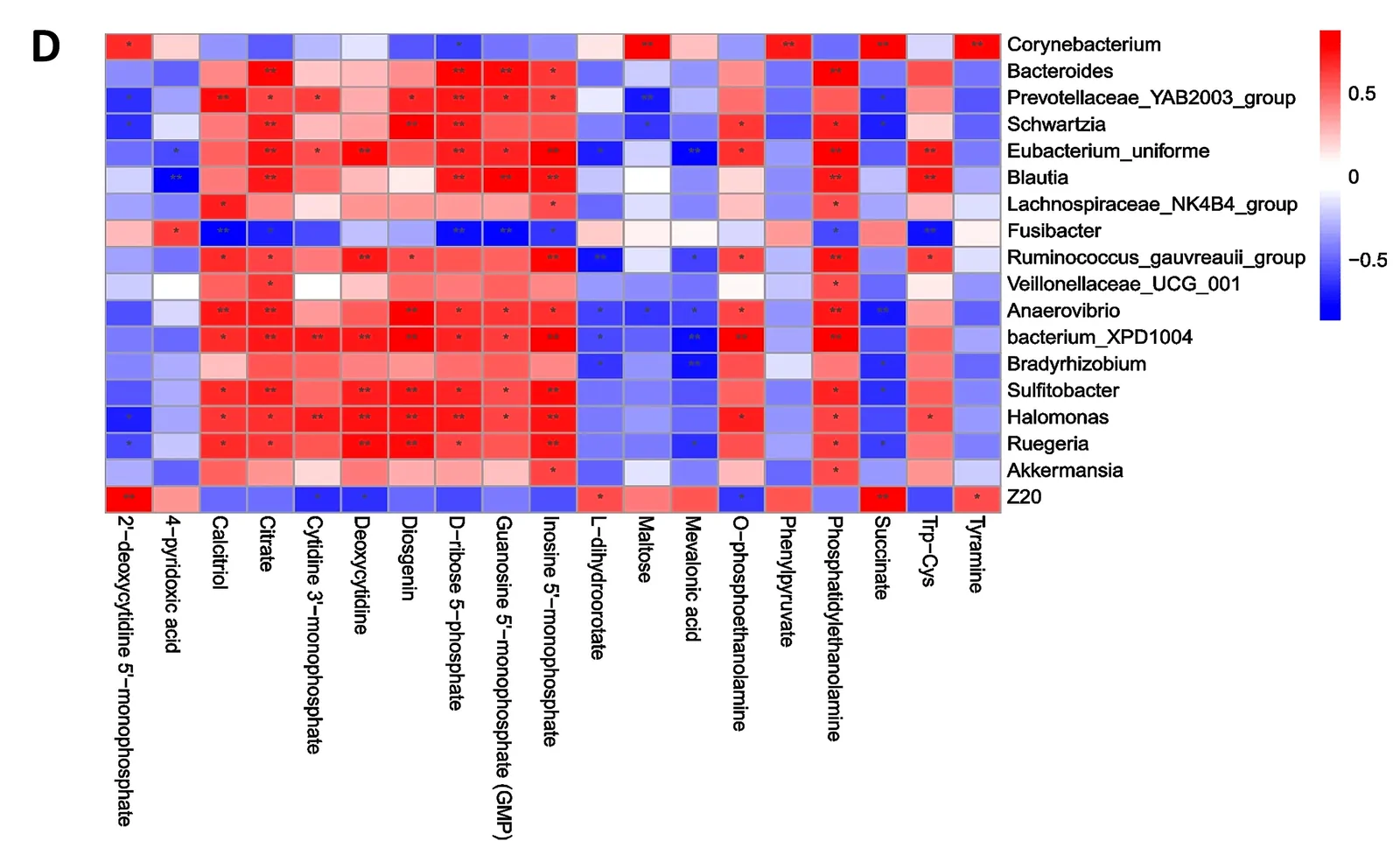
Differences in the gut microbiome and metabolic profiles of prepartum dairy cows with different body condition scores (BCSs). (**A**) Relative composition of the ruminal microbiota at the phylum and genus levels in cows with low-BCS (LBCS) and high-BCS (HBCS) groups. The charts illustrate differences in microbial community composition between the BCS groups. (**B**) Cladogram showing significantly different rumen microbiota between the LBCS and HBCS groups based on 16S rRNA gene sequencing data. (**C**) Abundances of significantly different bacterial genera between the LBCS and HBCS groups based on 16S rRNA gene sequencing data. Significant differences in panels B and C were identified using linear discriminant analysis effect size, with a linear discriminant analysis score > 2 and *p* < 0.05. (**D**) Correlation between significantly different microbes and metabolites based on Spearman’s correlation analysis. * indicates *p* < 0.05; ** indicates *p* < 0.01. Red and blue indicate positive and negative correlations, respectively (Adapted from [71], published under CC BY 4.0).

Differences in rumen and fecal microbial composition have been associated with body condition score (BCS) [71,72]. In the high-BCS (HBCS) group, genera within the phylum *Firmicutes* showed greater abundance and richness. Phyla such as *Firmicutes*, *Patescibacteria*, *Acidobacteriota*, *Euryarchaeota*, and *Desulfobacterota* were significantly more abundant in the HBCS group than in the low-BCS (LBCS) group (Figure 3A–C). At the genus level, *Anaerovibrio*, Veillonellaceae_UCG_001, *Ruminococcus gauvreauii*, *Blautia*, *Eubacterium*, Prevotellaceae_YAB2003_group, *Schwartzia*, and *Halomonas* were significantly more abundant in the HBCS group (Figure 3A). The tricarboxylic acid cycle, which is involved in carbohydrate metabolism, displayed a significant enrichment trend, accompanied by a notable increase in citrate abundance in the HBCS group (Figure 3D). This increase was significantly and positively correlated with the differential bacterial genera [71]. 16S rRNA sequencing revealed that the LBCS group had significantly greater abundances of *Clostridiaceae*, *Erysipelotrichales*, *Erysipelotrichaceae*, and *Turicibacter*, while cows with normal BCS had higher abundances of *Clostridiales*_vadinBB60_group, UCG-010, *Bacteroidaceae*, *Ruminococcaceae*, *Paludibacteraceae*, *Alistipes*, and *Bacteroides* [72]. These differences in ruminal microbial composition and metabolite profile may influence metabolic adaptation during the transition to lactation. These microbial differences may reflect altered substrate availability, energy balance, or host metabolic status and could contribute to subsequent lactation performance through changes in fermentation and metabolic signaling. Further longitudinal studies are needed to establish whether these prepartum microbial signatures predict milk yield, milk composition, or metabolic health during lactation.

## 7. Impact of the Gut Microbiota on Milk Yield

The gut microbiota, especially the ruminal microbial community, plays a central role in milk production. These microbes ferment indigestible plant materials into metabolites that can be absorbed and used as precursors for milk components [7,8,10]. One of the primary ways gut microbes impact milk yield is through the fermentation of dietary fiber and carbohydrates into VFAs, which serve as major energy substrates for the host. The principal VFAs produced are acetate, propionate, and butyrate, each of which contributes differently to milk biosynthesis [73,74]. Acetate and butyrate are especially important for de novo synthesis of milk fat in the mammary gland, while propionate is a major substrate for gluconeogenesis in the liver, thereby supporting lactose synthesis and, consequently, milk volume [73,74]. Studies have shown that cows with a greater abundance of acetate- and butyrate-producing bacteria tend to have higher milk fat content, while efficient propionate production is associated with improved energy balance and higher milk volume [75]. Furthermore, dietary strategies that promote fiber-degrading bacteria, such as *Ruminococcus* and *Fibrobacter*, have been linked to improved feed digestibility and VFA production, contributing to enhanced lactational performance [76].

Microbial protein synthesis is another important pathway through which the gut microbiota influences milk composition. Rumen microbes synthesize protein using nitrogen sources, including urea and ammonia, generating microbial biomass that serves as a primary amino acid source for the host following postruminal digestion. These amino acids support maintenance and milk protein synthesis. Efficient microbial protein synthesis improves nitrogen utilization and may support milk protein synthesis [74]. Additionally, specific bacterial genera, such as *Prevotella,* have been associated with ruminal protein metabolism and milk protein content [75].

Beyond energy and protein metabolism, the gut microbiota also influences milk production through effects on the immune system and systemic inflammation [77,78]. A balanced microbial community helps maintain gut barrier integrity and limits the translocation of endotoxins, such as LPS, into the bloodstream. When the gut barrier is compromised, as in the case of dysbiosis, LPS can enter systemic circulation and trigger inflammatory responses that may adversely affect milk yield and composition. Inflammatory cytokines are known to reduce feed intake, divert nutrients away from lactation toward immune functions, and impair mammary gland function [79,80]. Thus, maintaining microbial balance in the gut indirectly supports milk production by limiting metabolic stress and preserving nutrient availability for lactation.

Emerging research also highlights the influence of the gut microbiota during the transition period, which spans the three weeks before and after calving. This period is marked by a sudden increase in energy demand associated with the onset of lactation. Cows often experience negative energy balance during this period, which can alter microbial composition and activity. Dysbiosis during this critical window has been linked to metabolic disorders such as ketosis and fatty liver, which subsequently reduce milk yield [81]. Conversely, a well-adapted gut microbiota during this period promotes more efficient energy extraction from the diet and better metabolic adaptation, thereby contributing to improved milk output and stability of milk components [81].

Microbiota composition also varies among individual cows, and these differences have been linked to variation in lactational performance. For example, a study showed that even when rumen contents were exchanged between cows, recipient animals reestablished distinct microbial communities over time, indicating that host genetics and physiological characteristics influence microbial colonization. This host–microbiome interaction may partly explain why some cows outperform others under similar environmental conditions and feeding regimes [82].

Recent studies using metagenomic and metabolomic approaches have advanced our understanding of how gut microbes affect milk traits. A recent study found that the abundance of certain bacterial taxa correlated positively with milk fat and protein percentages, while greater microbial diversity and the presence of certain fiber-degrading and lactic acid-utilizing bacteria were associated with improved feed efficiency and higher milk solids [74]. Moreover, bacterial metabolites produced during rumen fermentation, including biohydrogenation intermediates (e.g., trans-10, cis-12 conjugated linoleic acid), can influence milk fat synthesis in complex ways, sometimes even leading to milk fat depression depending on dietary conditions [83].

Manipulation of the gut microbiota through dietary strategies has become an area of growing interest for improving milk production. Supplementation with direct-fed microbials (probiotics), such as *Saccharomyces cerevisiae*, *Lactobacillus*, and *Bacillus* species, as well as prebiotics such as yeast cell wall polysaccharides, can enhance ruminal fermentation, stabilize ruminal pH, and promote the growth of beneficial microbes. These interventions have been associated with increased feed intake, improved nutrient digestibility, and enhanced milk yield and composition [84]. Additionally, phytobiotics and essential oils have demonstrated antimicrobial properties that selectively suppress harmful bacteria and support beneficial populations in the rumen. These microbial shifts often improve VFA profiles and reduce methane production, thereby limiting energy losses and increasing the energy available for milk production [85].

## 8. Influence on Milk Composition

Microbial metabolites and microbial protein can contribute to the precursors required for milk component synthesis. Acetate and butyrate are important substrates for mammary fatty acid synthesis, whereas propionate contributes to hepatic gluconeogenesis and thereby supports glucose availability for lactose synthesis. However, observed associations between ruminal microbial taxa, VFA production, and milk traits should be interpreted as associations rather than evidence that changes in individual microbial genera directly increase milk yield or milk components. Dietary composition, energy balance, stage of lactation, and host metabolism can substantially modify these relationships.

### 8.1. Milk Fat

The pathways for de novo fatty acid synthesis in the mammary gland are largely supported by acetate and butyrate, which are derived from ruminal fermentation of dietary fiber [44]. The *F/B* ratio and the abundance of taxa such as *Butyrivibrio* and *Ruminococcus* have been linked to higher acetate production and milk fat yield in dairy buffaloes [37] and dairy cows [46]. Dietary interventions, such as supplementation with rice bran oil, can alter ruminal microbial composition and VFA profiles, thereby modifying milk fatty acid composition and increasing the proportion of beneficial fatty acids such as conjugated linoleic acid (e.g., enriched conjugated linoleic acid) [86].

### 8.2. Milk Protein

Efficient microbial nitrogen utilization, supported by cellulolytic and proteolytic microorganisms, contributes to the supply of amino acids for milk protein synthesis [87]. In dairy buffaloes, the abundance of genera such as *Prevotella* and *Bacteroides* was associated with serum concentrations of metabolites involved in protein biosynthesis in high-yielding animals [36]. Rumen microbial composition and metabolic activity are associated with nutrient utilization and milk production traits, underscoring the potential of dietary management to influence microbial functions and host productivity [36].

### 8.3. Milk Vitamins and Bioactive Compounds

Gut microbes are also responsible for the synthesis of vitamins (notably B12) and other bioactive compounds that contribute to milk’s nutritional profile. High ruminal concentrations of vitamin B12 have been associated with increased abundance of *Prevotella*, while *Bacteroidetes*, *Ruminiclostridium*, and *Butyrivibrio* have been associated with lower vitamin B12 concentrations (Table 3). These associations suggest that targeted manipulation of the rumen microbiota may eventually provide an opportunity to influence the vitamin B12 content of milk, although this has not been tested experimentally [67]. Such relationships should therefore be considered preliminary and require experimental validation to establish causality and the extent of transfer to milk.

### 8.4. Lactose

The gut microbiota contributes to lactose synthesis by influencing feed digestion and nutrient absorption. Microbial fermentation of complex carbohydrates produces VFAs, including acetate, propionate, and butyrate, which serve as important energy sources and metabolic substrates for the lactating cow [58,62]. Among these VFAs, propionate is particularly important for lactose synthesis because it serves as a major substrate for hepatic gluconeogenesis. Accordingly, the abundance of propionate-producing bacteria, including certain *Prevotella* and *Bacteroides* spp., has been associated with milk yield [88,89]. Because milk lactose concentration is relatively constant, increased glucose supply is expected to affect milk volume more than lactose percentage.

**Figure 4:**
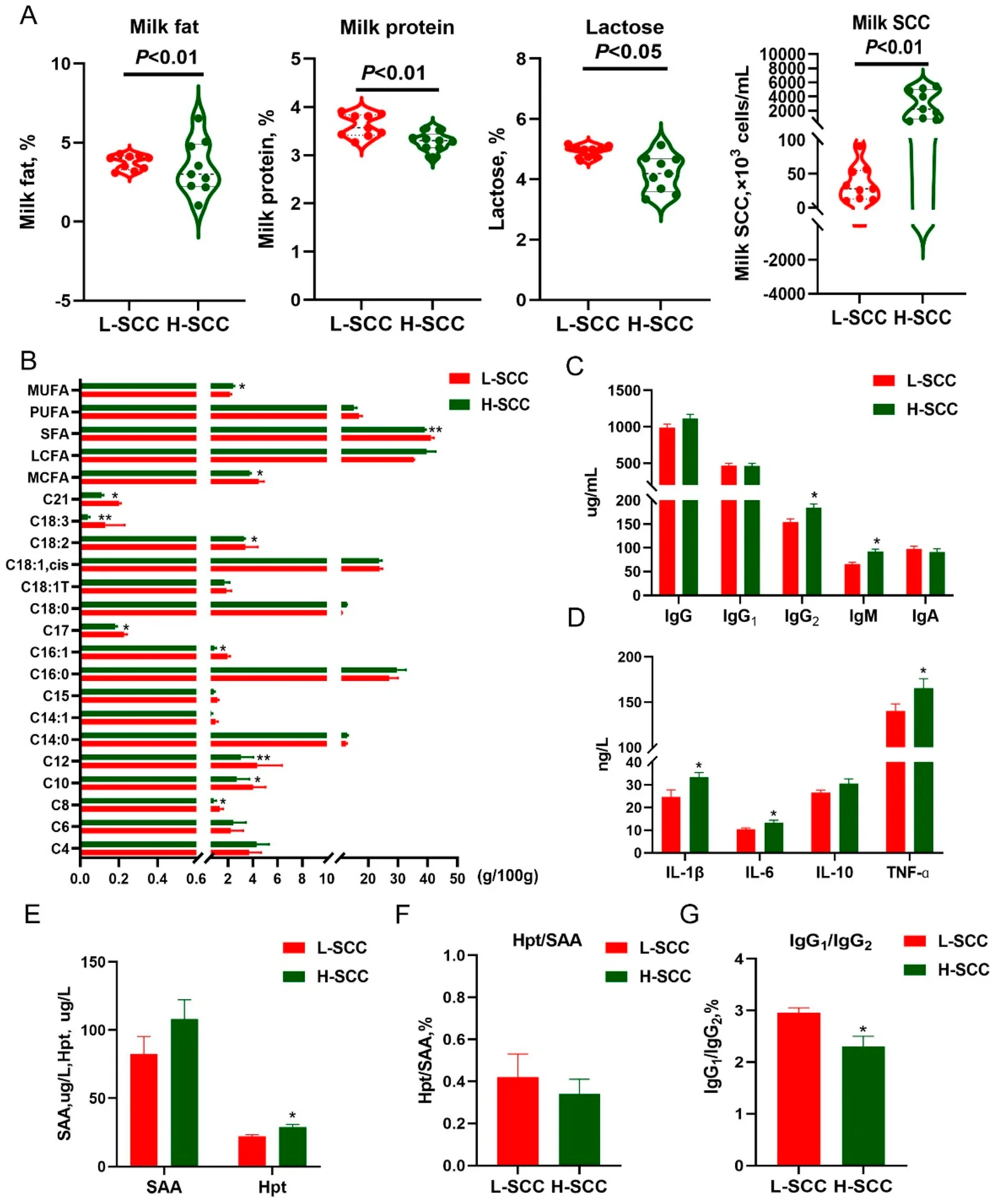
Milk composition and serum immune and inflammatory parameters in dairy cows with low-SCC (L-SCC) and high-SCC (H-SCC). The figure illustrates differences in milk composition and serum immune and inflammatory parameters between the two SCC groups. (**A**) Milk composition (milk fat, %; milk protein, %; lactose, %; milk SCC, ×10^3^ cells/mL). (**B**) Milk fatty acid composition, including caprylic acid (C8:0), capric acid (C10:0), palmitoleic acid (C16:1, cis-9), heptadecanoic acid (C17:0), octadecadienoic acid (C18:2, cis-9,12), heneicosanoic acid (C21:0), lauric acid (C12:0), and linolenic acid (C18:3, cis-9,12,15) in L-SCC and H-SCC cows. (**C**) Serum immunoglobulin concentrations. (**D**,**E**) Serum cytokine concentrations. (**F**) Hpt/SAA ratio. (**G**) IgG1/IgG2 ratio. Data are presented as mean ± SD. * indicates *p* < 0.05; ** indicates *p* < 0.01 between the L-SCC and H-SCC groups (Adapted from [90], published under CC BY 4.0).

A recent study reported differences in ruminal microbial composition, fermentation products, milk composition, and immune-related parameters between cows with low and high somatic cell counts (SCC) [90]. Compared with the low-SCC group, high-SCC cows showed lower milk fat, protein, and lactose concentrations (Figure 4A) and reduced ruminal fermentation products, together with higher abundances of *Bacteroidetes*, *Firmicutes*, *Lachnospiraceae*, *Prevotella*, and *Ruminiclostridium* [90]. The H-SCC group had lower proportions of saturated fatty acids (SFAs) (*p* < 0.01) and medium-chain fatty acids (*p* < 0.05) than the L-SCC group. Long-chain fatty acids and polyunsaturated fatty acids did not significantly differ between the groups. Although IgA concentrations did not differ significantly between the groups (Figure 4C), IgG2 and IgM concentrations were significantly higher in the H-SCC group than in the L-SCC group (*p* < 0.05). The H-SCC group demonstrated significantly higher levels of IL-1β, IL-6, TNF-α, and haptoglobin (Hpt) than the L-SCC group (*p* < 0.05), indicating systemic inflammation. However, no significant variations in IL-10 or serum amyloid A (SAA) were observed between the two groups (Figure 4D,E). Furthermore, the IgG1/IgG2 ratio was significantly lower in the H-SCC group than in the L-SCC group (*p* < 0.05; Figure 4G) [90]. These findings indicate associations between ruminal microbiota and SCC-related metabolic and milk-composition differences; however, further study is needed to validate whether microbial changes contribute to increased SCC or occur as a consequence of altered host physiology. In contrast to the report in [48], this study found greater *Prevotella* abundance in H-SCC cows. Therefore, *Prevotella* abundance should not be interpreted as uniformly protective, and differences among studies may reflect variation in host status, diet, microbial community structure, or study design. The conflicting findings also highlight the need for longitudinal and mechanistic studies to determine the direction and biological significance of this association.

## 9. Strategies to Manipulate the Cattle Gut Microbiota

The gut microbiota of cattle plays a pivotal role in nutrition, immunity, disease resistance, and greenhouse-gas emissions. Emerging pressures such as restrictions on antibiotic growth promoters (AGPs), the need to reduce methane emissions, and the demand for sustainable livestock production have accelerated the development of microbiome-based interventions. This section details contemporary strategies for manipulating the cattle gut microbiota, spanning bioactive feed additives, microbial engineering, and precision interventions.

### 9.1. Probiotic Supplementation

Probiotic supplementation modulates the gut microbiome through the administration of live beneficial microbes. *Lactobacillus*, *Bifidobacterium*, *Bacillus*, *Enterococcus*, and *Saccharomyces cerevisiae* are the most studied taxa and have been reported to reduce diarrhea and improve early growth performance in calves, although results vary among products and studies [91]. In adult cattle, direct-fed microbials, including yeast and mixed bacterial preparations, have been evaluated for effects on nutrient digestibility, ruminal fermentation, and lactation performance [86,91]. These effects are dependent on the specific strain, dose, formulation, and administration conditions and should not be generalized to all members of a genus.

In cows with elevated SCC, dietary lactic acid bacteria and yeast lowered milk SCC and milk inflammatory markers and reduced the relative abundance of *Enterococcus* and *Streptococcus* in milk [5]; effects on clinical mastitis incidence were not assessed. Other probiotics, including *Saccharomyces cerevisiae*, *Megasphaera elsdenii*, *Butyrivibrio fibrosolvens*, and various *Lactobacillus* and *Bifidobacterium* strains, can stabilize ruminal pH, promote SCFA production, and antagonize pathogens [77]. Supplementation of newborn calves with the probiotic *Limosilactobacillus reuteri* SW23 enhanced immune responses, which led to increased performance and gut health and reduced the incidence of diarrhea [92]. In a mouse model, *Clostridium tyrobutyricum* protected against *Staphylococcus aureus*-induced mastitis by improving the integrity of the blood–milk barrier through the production of butyrate [20]. These immunomodulatory microbes are being investigated as potential alternatives to antibiotics in reducing antimicrobial resistance while improving health and productivity.

### 9.2. Prebiotics

Prebiotics are defined as substrates that are selectively utilized by host microorganisms, conferring a health benefit. Common examples include inulin, fructooligosaccharides (FOS), galactooligosaccharides (GOS), and xylooligosaccharides. In ruminants, inulin supplementation has been reported to reduce ruminal ammonia and methane production, increase microbial protein synthesis, and improve calf weight gain [93]. Carbohydrate-based prebiotics (e.g., FOS and GOS) have been widely used in several studies. Supplementation with GOS has been shown to reduce *Citrobacter rodentium* adherence to epithelial cells in vitro; however, in mice, GOS did not reduce colonic adherence or systemic dissemination, despite reducing colonic disease severity [94]. Probiotic–prebiotic combinations may have complementary or synergistic effects, but such synergy is dependent on the specific strains, substrates, doses, and experimental context. In buffalo calves, combinations of host-specific probiotics and prebiotic substrates increased the abundance of *Prevotella*, improved feed efficiency and growth, and reduced the incidence of diarrhea [95]. Similarly, FOS combined with *Lactobacillus plantarum* CRD-7 was associated with fewer gastrointestinal disorders in buffalo calves [96]. Mechanistically, prebiotics undergo saccharolytic fermentation in the rumen and colon, generating SCFAs, particularly propionate and butyrate, which contribute to energy metabolism and epithelial barrier maintenance [97,98].

### 9.3. Synbiotics

Synbiotics combine probiotics and prebiotics with the aim of achieving complementary or synergistic effects. In buffalo calves, synbiotic administration has been associated with improved gut health, immunity, and growth performance [96]. In Murrah buffalo calves, a synbiotic formulation containing 6 g of FOS and *L. plantarum* CRD-7 enhanced immune function, antioxidant enzyme activity, and nutrient digestibility. It also altered the fecal microbiota and reduced the likelihood of diarrhea. Compared with the control group, the treated group exhibited greater crude protein digestibility and average daily growth (*p* < 0.05) [96]. Similar findings were observed in another study [99]. By combining live microorganisms with substrates that support their activity, synbiotics may serve as a promising alternative to antibiotic growth promoters for maintaining intestinal stability and improving livestock productivity. A synbiotic formulation containing *Cichorium intybus* root powder with *L. acidophilus* NCDC15 and *L. reuteri* BFE7 enhanced growth performance in Murrah buffalo calves by modifying certain gut health indices [100].

### 9.4. Postbiotics and Parabiotics

Beyond live microbial preparations, postbiotics have emerged as potential microbiome-based interventions. According to the ISAPP consensus, a postbiotic is a preparation of inanimate microorganisms and/or their components that confers a health benefit on the host, with or without metabolites; purified metabolites alone are not postbiotics [101]. Inactivated whole-cell preparations, sometimes termed parabiotics, fall within this definition [102]. Their stability, chemical composition, and shelf life vary according to the specific preparation and processing conditions. Although their research in cattle is limited, studies in other species and limited data in beef cattle suggest potential benefits for gut health, pathogen suppression, and feed efficiency without the risks associated with live cultures [103,104]. In Friesian cows, two postbiotics derived from wheat bran were evaluated from calving through the first two months of lactation. Postbiotic supplementation during late pregnancy and early lactation increased voluntary DMI and apparent total tract nutrient digestibility. Supplementation was also associated with increased colostral immunoglobulin concentrations and higher milk fat and protein yields [4].

### 9.5. Rumen Transfaunation and Fecal Microbiota Transplant (FMT)

Veterinary practice has long used rumen transfaunation, in which rumen contents from healthy animals are administered to recipients to help restore ruminal microbial function and digestive flora [105]. This approach is used to help re-establish ruminal microbial function in animals with digestive disorders, although controlled efficacy data remain limited. Modifying the rumen microbiome early in life has the potential to improve long-term productivity, health, and calf development [106]. Early-life FMT has also been investigated as a strategy for influencing gut microbial development in neonatal calves. By lowering the pro-inflammatory response and raising the antioxidant capacity, FMT therapy mitigated the adverse effects of weaning stress [107]. The scalability and biosecurity of transfaunation and FMT, including the risk of pathogen transmission, require further attention [105].

### 9.6. Dietary Additives for Methane Management

Amid climate concerns, dietary strategies targeting ruminal methanogenic archaea are emerging for reducing enteric methane production. In vivo studies have revealed that red seaweed *Asparagopsis taxiformis* has been reported to strongly reduce methane production in rumen fermentation of dairy cows, particularly at 0.50% of the substrate. The treatment also altered rumen fermentation, with lower acetate and higher propionate and butyrate levels. However, these findings cannot be directly extrapolated to feed intake or milk production in live dairy cows, and animal-level effects require in vivo validation [108]. The long-term ecological and production impacts also remain under evaluation. Enzymatic supplements, tannins, and carbohydrases also modify rumen microbiota [109]. Tannins can bind dietary proteins and have antimicrobial activity, while feed enzymes can improve fiber breakdown [109]. In grazing Nellore steers, a phytogenic blend containing hydrolyzable tannins, carvacrol, and cinnamaldehyde reduced the ruminal proportion of valerate and the abundance of several *Firmicutes* and *Verrucomicrobia* taxa, but did not improve nitrogen retention [110]. Condensed tannins can reduce ruminal proteolysis, thereby increasing the proportion of dietary nitrogen available for post-ruminal digestion and absorption, lowering urinary excretion, and improving overall nitrogen retention [110].

### 9.7. Microbial and Molecular Innovations

CRISPR-based engineering has been proposed as a potential strategy to modify methanogenic archaea, including *Methanobrevibacter ruminantium*, to redirect carbon metabolism toward acetate production [111]. Although its feasibility and effects on rumen fermentation remain to be experimentally established, it may offer a future route to methane mitigation. Machine learning (ML) is increasingly being leveraged to select probiotic strains and design personalized interventions. Recent studies have applied ML to link microbiome changes following *L. reuteri* treatment with calf health outcomes, thereby enhancing the accuracy of probiotic strain screening [112].

### 9.8. Environmental and Antibiotic Management

The move away from AGPs is necessary. Antibiotics can alter commensal microbiota, causing gut dysbiosis [113]. In Japanese Black calves, chlortetracycline disrupted microbiome homeostasis, reducing butyrate-producing bacteria while increasing methanogens [26]. Such imbalance can impair immunity, growth, and sustainability. Monitoring pasture-induced dysbiosis, such as that associated with fescue toxicosis, through microbiome profiling enables early detection. Genomic studies have revealed shifts towards resistant taxa under toxin stress, supporting the development of potential biomarkers [114]. Implementing strict hygiene protocols in housing facilities, milking parlors, and calf-rearing areas can considerably reduce the incidence of diseases such as mastitis, thereby minimizing the need for antibiotics [115]. Researching and implementing non-antibiotic alternatives for disease prevention and treatment, such as vaccines, probiotics, and other therapeutic interventions, can further reduce reliance on antibiotics [116].

### 9.9. Dietary Strategies

**Figure 5:**
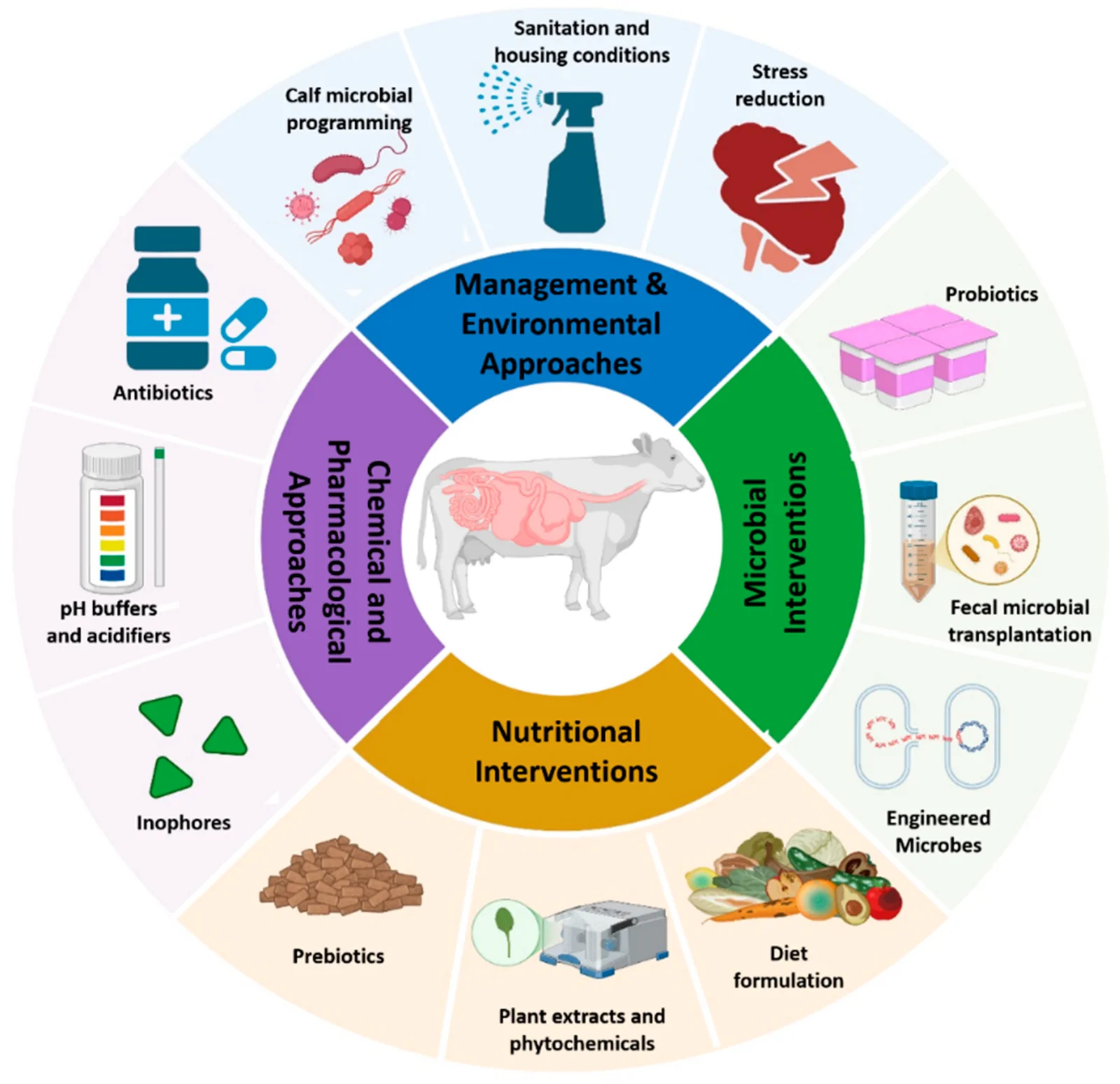
Strategies for manipulating gut microbiota in cattle. The figure was created using BioRender software.

Rations supplying highly degradable protein (e.g., lupin) can lead to excess ammonia production if they are not adequately balanced with rapidly fermentable energy sources, such as barley starch [87]. Synchronizing energy and nitrogen release enhances microbial uptake, thereby preventing ammonia accumulation and reducing urinary nitrogen excretion. Supplementation with rumen-protected essential amino acids (e.g., methionine and lysine) can help meet animal requirements more precisely and improve nitrogen-use efficiency by reducing the need for high dietary crude protein inputs [61]. Microbial urease activity facilitates the utilization of recycled urea-N by converting urea to ammonia, which, when sufficient fermentable energy is available, can be efficiently assimilated into microbial protein [117] (Figure 5).

## 10. Conclusions

The integration of multi-omics technologies, including metagenomics, metabolomics, and transcriptomics, is revealing associations among microbial taxa (e.g., *Muribaculaceae*, *Moryella*, and *Weissella*), metabolites, and the upregulation of immune genes (e.g., *TLR4*, *LY96*, and *MBL2*) in cattle [22,33]. These studies advance understanding of the gut–mammary axis and how it may be harnessed to improve cattle health, productivity, and product quality.

Manipulation of the cattle gut microbiota is an active area of research in sustainable livestock production. Probiotics, prebiotics, synbiotics, postbiotics, and advanced genomic tools are collectively expanding opportunities to improve animal health and productivity while reducing environmental impacts. Tailoring these interventions to host species, management systems, and local challenges can maximize their benefits. With multidisciplinary support, including policy harmonization and farmer education, microbiome optimization may become an integral component of ethical, efficient, and climate-friendly cattle production.

In conclusion, manipulating the gut microbiota through targeted nutritional and microbial interventions represents a potential strategy to improve milk production, enhance milk quality, and promote overall cow health. However, a deeper mechanistic understanding of microbiome–host interactions is needed to translate microbiome research into practical applications. In particular, strain-level evidence for gut-to-mammary translocation in cattle, prospective validation of microbial markers of mastitis risk, and controlled intervention trials that report clinical mastitis incidence and lactation outcomes are required. Such knowledge will be instrumental in developing precision livestock farming strategies that align with animal welfare, environmental sustainability, and economic viability in the dairy sector.

## Figures and Tables

**Table 1:** Key relationships between gut microbiota structure/function and immune modulation in mammals.

Microbial Taxa/Group	Mechanism of Immune Modulation	Immune Outcomes	Research Insights	Reference
*Prevotella* spp.	Production of SCFAs (e.g., acetate, propionate); antigen presentation; interaction with TLRs; modulation of the cytokine milieu	Proposed to support anti-inflammatory responses and epithelial barrier integrity; effects are strain- and host-dependent	High abundance has been linked to improved gut homeostasis and reduced systemic inflammation	[23,24,36,37]
*Bifidobacterium* spp.	Strengthen mucosal layer, increase IgA production, inhibit pathogen colonization	Promote mucosal immune maturation and protection against infections	Early-life colonization aids immune development; commonly included in bovine probiotic strategies	[24,38,39]
*Lactobacillus* spp.	Production of lactic acid/bacteriocins; modulation of cytokine expression and TLRs; signal via MAMP receptors	Stimulate innate immunity; reduce pro-inflammatory cytokines	Improve mucosal health and resistance to enteric pathogens, particularly in calves	[38,39,40]
*Faecalibacterium prausnitzii*	Butyrate production; support of epithelial barrier integrity; IL-10 induction	Anti-inflammatory cytokine secretion (e.g., IL-10); enhanced barrier function	Indicator of healthy gut; butyrate production is central to tolerogenic immune balance	[23,24,39]
*Clostridium* clusters IV and XIVa	Promote differentiation and expansion of regulatory T cells via SCFA (butyrate) and other metabolites	Suppression of gut inflammation and promotion of immune tolerance	Contribute to mucosal tolerance, important for immune homeostasis and prevention of immune-mediated disease	[37,39,41]
*Ruminococcus* spp.	Fiber fermentation and SCFA production; promote mucus production; prevent pathogen colonization	Indirectly promote immune tolerance; strengthen epithelial defense	Fiber fermentation sustains commensal-driven immune education	[23,36,39]
*Bacteroides* spp.	Produce immunomodulatory polysaccharides (PSA); modulate TLR signaling; SCFA production	Enhance epithelial defense; regulate pro-/anti-inflammatory balance	PSA promotes Treg induction; balance with Firmicutes may influence immune outcomes	[23,37,39]
Pathogenic *E. coli* strains	Disrupt epithelial barriers; upregulate pro-inflammatory cytokines (IL-6 and TNF-α)	Induce innate immune responses; cause inflammation, diarrhea, and sepsis	Overgrowth is associated with calf diarrhea and disease outbreaks	[24,42,43]
*F/B* ratio	Influence on energy extraction, SCFA production, modulation of immune cell populations	Associated with differences in systemic immune status and metabolic inflammation; effects on barrier integrity are context-dependent	Variation in the ratio is associated with dietary and environmental factors affecting immune homeostasis	[23,39,44]

**Table 2:** Key relationships between gut microbiota structure/function and mastitis in cattle.

Microbial Taxa/Gut-Derived Component	Mechanism of Action & Gut–Mammary Axis Crosstalk	Effect on Mastitis Risk	Key Insights and Omics Evidence	Reference
*Prevotella* spp.	Produce SCFAs (propionate and acetate); proposed to support the mucosal barrier and anti-inflammatory signaling	Associated with lower inflammation in some studies; direction inconsistent across studies	Higher abundance in the gut has been associated with reduced mastitis severity and improved immune homeostasis; positively linked to serum SCFAs and host metabolic health.	[48]
*Butyrivibrio* spp. and *Faecalibacterium prausnitzii*	Produce butyrate and other SCFAs; support Treg cells and maintain epithelial integrity; anti-inflammatory metabolites reach systemic circulation	Reduce inflammation in the mammary gland	Butyrate-producing taxa contribute to host immune tolerance; lower abundance has been observed in cows during and preceding mastitis episodes; enriched in high yield/healthier animals.	[49,50]
*Lactobacillus* spp. and *Bifidobacterium* spp.	Enhance gut barrier; compete with pathogens; modulate IL-10, IL-6, and TNF-α; impact systemic immunity through metabolites/cytokines	Improve resistance, reduce SCC	Oral supplementation with these genera alters gut microbiota, improves immune markers, and has been associated with lower SCC and milk inflammatory markers; effects on clinical mastitis incidence remain to be established.	[51]
Firmicutes/Bacteroidetes (*F/B*) ratio	May influence SCFA type and quantity, barrier function, and mucosal and systemic immune balance	Associations with mastitis risk vary across studies and experimental conditions	Variation in the *F/B* ratio has been associated with mastitis-related microbial and inflammatory changes	[32]
*Pathogenic E. coli* and *Enterobacteriaceae*	Pathogen overgrowth in the gut; translocation of LPS into systemic circulation triggers inflammation in the mammary gland	Major facilitator of coliform mastitis	Evidence, mainly from mouse models, supports entero-mammary transmission and LPS-driven inflammation; gut dysbiosis increases the risk of environmental mastitis outbreaks.	[43,52,53]
Lipopolysaccharide (LPS)	LPS crosses compromised gut barrier, enters bloodstream and activates TLR4 pathway in mammary tissue	Exacerbates clinical or subclinical mastitis	Gut dysbiosis and impaired gut barrier integrity increase circulating LPS, thereby initiating or aggravating mastitis.	[43,53]
Gut–Mammary Axis (Functional Crosstalk)	Gut-derived metabolites, cytokines, and immune cell “education” influence mammary immune readiness and inflammation	Proposed regulatory pathway	Multi-omics indicate that shifts in gut and ruminal microbiota alter systemic metabolic and immune signaling that is associated with mastitis susceptibility, severity, and mammary gland health.	[36,53]

**Table 3:** Key microbial taxa and their possible impact on milk production and composition.

Microbial Taxon/Function	Main Effect	Impact on Milk Production	Animal Species	Study Type	Reference
*Prevotella* spp.	Fiber and carbohydrate fermentation; VFA and protein synthesis	Might be associated with milk yield, fat and protein content, and vitamin B12 concentrations	Dairy cattle	Observational microbiome–milk trait association	[36,44,67]
*Butyrivibrio* spp.	Butyrate production and lipid metabolism	Might be associated with milk fat content and fatty acid profile	Dairy cattle	Observational microbiome–milk trait association	[36,44]
*Ruminococcus* spp.	Cellulose degradation; acetate and butyrate production	Might be associated with milk yield and fat content	Dairy cattle	Observational microbiome–milk trait association	[36,44]
*Bacteroides*/*Barnesiella* spp.	Carbohydrate and lipid metabolism	Might be associated with milk yield, fat, and metabolites	Dairy cattle	Observational microbiome–metabolite association	[36]
Methanogens	Methanogenesis; H_2_ consumption	Might be associated with lower milk yield (when abundant)	Dairy cattle	Observational microbiome–production association	[36]
*Bacteroidetes*, *Ruminiclostridium* (high abundance)	Associated with lower ruminal vitamin B12 concentrations	Might be associated with lower vitamin B12 concentrations	Dairy cattle	Observational microbiome–milk trait association	[67]

## Data Availability

No new data were created or analyzed in this study. Data sharing is not applicable to this article.
